# Diagnostic Pitfalls in Obturator Hernia: A Case Report and Literature Review

**DOI:** 10.7759/cureus.110420

**Published:** 2026-06-07

**Authors:** Alexis Genaro Ortiz Altamirano, Allan M Gloria-Rivas, Raul Rodrigo Jara Gutierrez, Emmanuel Melo Hernandez, Alan Brian Perez Garcia

**Affiliations:** 1 General Surgery, General Hospital Institute for Social Security and Services for State Workers (ISSSTE) Dra. Matilde Petra Montoya Lafragua, Mexico City, MEX; 2 Surgery, American College of Surgeons, Chicago, USA

**Keywords:** case report, fecal peritonitis, geriatric surgery, obturator hernia, richter’s hernia, small bowel obstruction

## Abstract

Obturator hernia is a rare clinical entity that predominantly affects elderly, thin women due to anatomical factors such as a wider pelvis and the loss of preperitoneal fat. Despite its classic description, the preoperative diagnosis remains a major challenge due to its deep location and non-specific presentation as a mechanical small bowel obstruction. This report describes the case of a 66-year-old female whose history of prior abdominal surgeries created a diagnostic bias toward adhesive obstruction, resulting in an initial period of conservative management. The most significant clinical signs were absent, leading to a delay in obtaining advanced imaging. Computed tomography eventually identified a right-sided obturator hernia complicated by intestinal perforation. During emergency laparotomy, fecal peritonitis and a friable ileal loop with multiple perforations were identified, necessitating a significant bowel resection. Due to the gross contamination of the surgical field, the defect was repaired using a primary suture technique to minimize the risk of infection associated with prosthetic materials. The patient’s fatal outcome underscores that the prognosis of this condition depends primarily on the timing of the diagnosis and prompt surgical intervention. High clinical suspicion in geriatric patients with intestinal obstruction, even in those with a history of prior surgery, is fundamental to reducing the mortality associated with this condition. The choice of primary repair remains a safe and necessary strategy in the presence of fecal contamination to avoid complex postoperative complications.

## Introduction

Obturator hernia is a rare but life-threatening type of abdominal wall hernia, accounting for approximately 0.07% to 1% of all cases [1]. Anatomically, the protrusion occurs through the obturator canal and mainly affects elderly, thin, and multiparous women, a demographic often referred to as the “little old lady’s hernia” [2], because factors such as a wider pelvis and a more triangular obturator canal opening with a larger transverse diameter can increase the risk of developing an obturator hernia [3]. It is more common on the right side than the left because the sigmoid colon may prevent herniation by lying in front of the left obturator foramen [4]. Due to its deep anatomical location and non-specific symptoms, it is associated with significant delays in diagnosis [5].

The preoperative diagnosis remains a significant challenge for surgeons. The Howship-Romberg sign, pain in the medial thigh worsened by hip extension or internal rotation, appears in fewer than half of patients [2,6]. As a result, most cases are initially mistaken for simple mechanical bowel obstructions, often leading to delayed surgery and higher rates of bowel resection [1,5].

Currently, contrast-enhanced computed tomography (CT) is the gold standard for diagnosis [5]. Early detection through CT is inversely linked to perioperative mortality. It is key for identifying complications such as Richter-type strangulation or perforation [6]. It is associated with significant delays in diagnosis and high mortality rates, ranging from 12% to 25% [5].

As many factors can distract when dealing with these types of patients, making it difficult to detect them, we believe it is important to focus on the diagnostic approach.

## Case presentation

A 66-year-old female presented to the emergency department with a four-day history of diffuse, spasmodic abdominal pain, rated at 7/10 on the visual analog scale, radiating to the inner thigh and worsening with movement, accompanied by constipation and an inability to eliminate gas. Her medical history was significant for dyslipidemia, an open left inguinal hernioplasty 30 years prior, and a total abdominal hysterectomy 15 years ago due to uterine leiomyomatosis. A body mass index of 19 kg/m² and a nutritional status assessed using the two-point malnutrition screening tool indicated a high risk of malnutrition.

During the first visit (on the fourth day after the onset of symptoms), the patient received conservative treatment consisting of a bowel-cleansing enema and symptomatic treatment. She reported that the medical staff indicated she did not meet the criteria for hospitalization, after which she was discharged. However, she returned on day eight due to persistent bowel obstruction, feculent vomiting, and total intolerance to oral intake. On physical examination (day nine), the patient exhibited a severely distended abdomen with hyperactive bowel sounds with a metallic quality. Laboratory results revealed leukocytosis with neutrophilia, elevated C-reactive protein, and severe hyponatremia (Table 1). An abdominal X-ray showed dilated small bowel loops and multiple air-fluid levels (Figure 1).

**Table 1:** Laboratory findings. MCH: mean corpuscular hemoglobin; RDW: red cell distribution width; MHCH: mean corpuscular hemoglobin concentration; BUN: blood urea nitrogen; ALT: alanine transaminase; AST: aspartate transaminase; LDH: lactate dehydrogenase; INR: international normalized ratio

Test		Value	Unit	Reference range
Blood biometrics	Hemoglobin	14.6	g/dL	13.5–17
	Hematocrit	40.6	%	34–44
	Erythrocytes	4.6	×10^6^/mm^3^	4.5–5.5
	Platelets	327	×10^3^	140–400
	Leukocytes	15.1	×10^3^	4.5–11
	MCH	29.1	pg	28–33.5
	RDW	12	%	11.6–14
	MHCH	33	g/dL	28–34
	Lymphocytes	38	%	18–46
	Monocytes	11	%	0–12
	Basophils	1.2	%	0–4
	Eosinophils	0	%	0–1
	Neutrophils	91	%	50–70
Blood chemistry	Glucose	138	mg/dL	70–100
	Creatinine	1.12	mg/dL	0.59–1.04
	Urea	136	mg/dL	12.8–56
	BUN	54	mg/dL	5–24
Serum electrolytes	Sodium	116	mmol/L	136–145
	Potassium	3.8	mmol/L	3.5–5.2
	Chlorine	70	mmol/L	102–112
	Calcium	8.5	mg/dL	8.7–10.2
	Magnesium	3.7	mg/dL	2.07–3.33
	Phosphorus	3.46	mg/dL	2.3–6
Liver function tests	ALT	34	U/L	7–36
	AST	31	U/L	14–36
	Total bilirubin	1.5	mg/dL	0.22–1.5
	Direct bilirubin	0.4	mg/dL	0–0.5
	Albumin	3.2	g/dL	4–5.3
	Alkaline phosphatase	51	U/L	30–100
	Gamma-glutamyl transferase	120	U/L	10–50
	LDH	271	U/L	128–249
Acute-phase reactants	Procalcitonin	0.76	ng/mL	0.5–2.0
	C-reactive protein	12.3	mg/dL	0.2
Coagulation times	Prothrombin time	11.2	Seconds	10–14
	Thromboplastin time	32.9	Seconds	25–45
	INR	0.98	-	0.8–1.2

**Figure 1:**
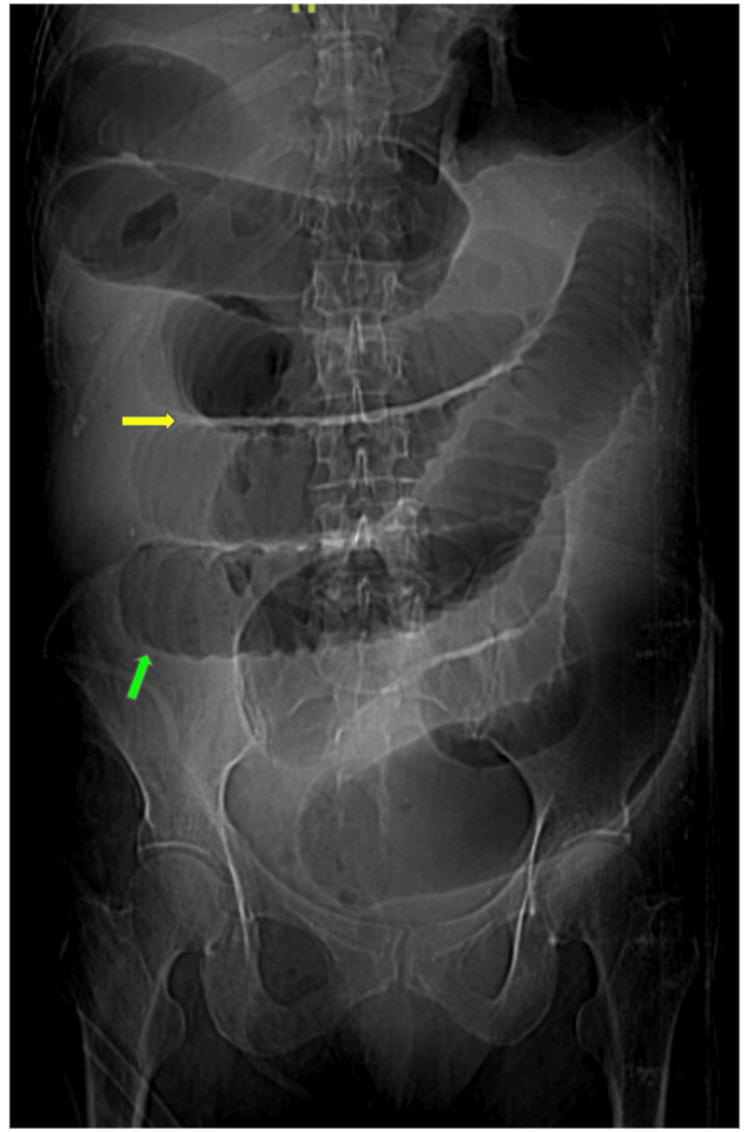
Plain abdominal X-ray showing significant small bowel distension with multiple air-fluid levels. The green arrow shows the stacked coin sign, and the yellow arrow shows wall edema.

As part of the standard protocol for all patients with intestinal obstruction and a history of previous surgeries, the following was administered as initial management, including nasogastric tube decompression, which drained 500 mL of gastrointestinal content. Due to the lack of clinical improvement after two days on a nasogastric tube and fluid output exceeding 500 mL, it was decided to perform a contrast-enhanced CT scan on the 10th day, as part of the preoperative evaluation. The CT revealed a mechanical small bowel obstruction with a transition point at the right obturator canal, where a loop of ileum was herniated, accompanied by free fluid and signs suggestive of intestinal perforation (Figure 2).

**Figure 2:**
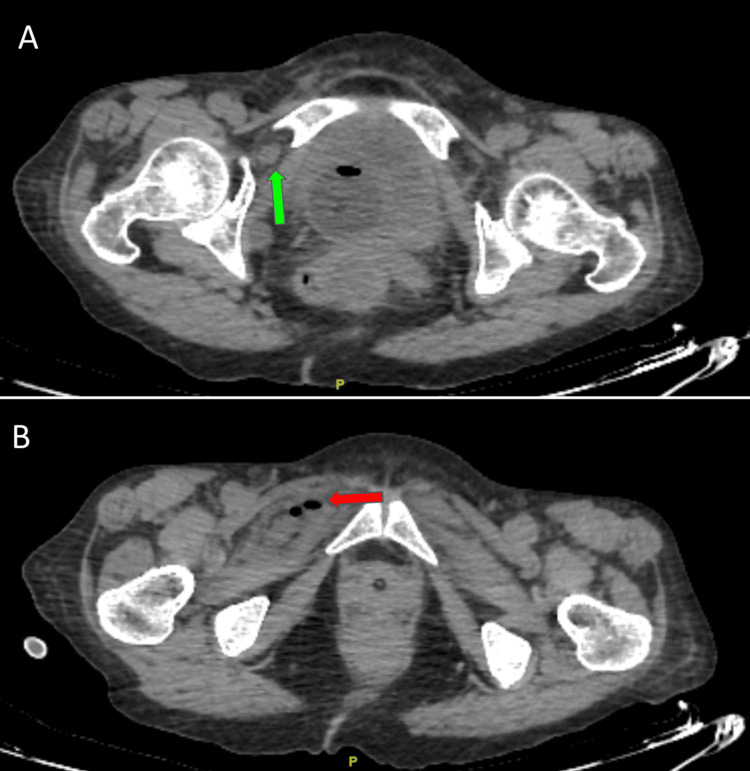
Computed tomography findings. (A) Transition point at the right obturator canal (green arrow). (B) A loop of the ileum is seen herniated between the pectineus and the external obturator muscles (red arrow).

Due to the slow clinical progression, a decision was made to perform an exploratory laparotomy. Surgical findings included fecal peritonitis and a Richter-type right obturator hernia involving a perforated segment of the ileum. Multiple additional perforations were identified proximally related to the duration of ischemia suffered by the intestinal wall (Figure 3).

**Figure 3:**
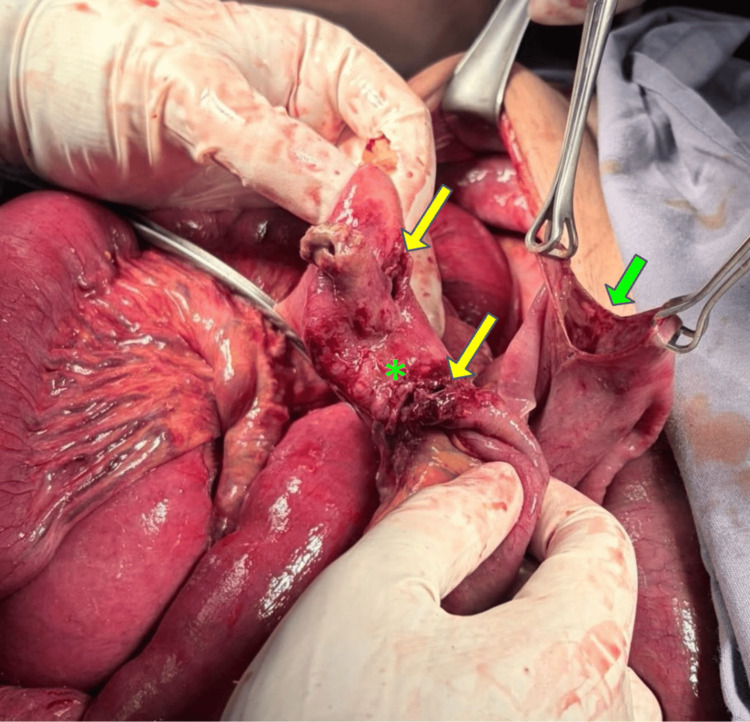
Intraoperative findings. The contents of the hernia following the exposure of the hernial sac (green arrow). An erythematous and friable loop of the small bowel is identified (asterisk), showing multiple perforations (yellow arrow) consistent with advanced ischemia and fecal peritonitis.

The surgical procedure consisted of a 50-cm ileal resection followed by a side-to-side functional stapled entero-enteric anastomosis, and a 50-cm resection of the ileum was performed, along with a mechanical side-to-side enteric anastomosis 110 cm from the Treitz angle and 50 cm from the ileocecal valve, as well as repair of serosal tears at 180 cm from the Treitz angle.

Postoperatively, the patient was transferred to the intensive care unit in septic shock, requiring mechanical ventilation and dual vasopressor support. Despite aggressive hemodynamic resuscitation and critical care management, the patient’s hemodynamic status continued to deteriorate, and the patient died on postoperative day after the intervention.

## Discussion

Obturator hernia is a rare but significant entity. Although a low incidence of inguinal hernia has already been mentioned [7], as our hospital does not have an epidemiological report, our incidence is not documented. Our patient, a 66-year-old woman, fit this demographic profile, as she had a low BMI and was at risk for malnutrition, as previously noted. The loss of preperitoneal fat due to age or emaciation facilitates herniation through the obturator canal [8,9]. As seen in this case, it is a major cause of small bowel obstruction, presenting in this manner in up to 63-93% of patients [1,3,8].

Diagnosis is notoriously difficult because symptoms often mimic other causes of obstruction. In addition, functional causes such as electrolyte imbalance should always be ruled out [10]. While the Howship-Romberg sign is highly suspected clinically, its sensitivity is only 27% to 56% [1,11]. In our case, the absence of this sign, combined with the patient’s history of prior inguinal and abdominal surgeries, initially led the surgical team to suspect adhesions. Second, our patient had severely low sodium levels, which caused ileus. This misdiagnosis led to an initial conservative treatment, as recommended by guidelines for intestinal obstruction due to adhesions [10], a common error in patients with a history of surgery. CT is the gold standard for diagnosis. As suggested in the literature, early CT scanning is inversely associated with mortality [8,11]; in our patient, the delayed CT scan confirmed not only the hernia but also the catastrophic progression toward perforation.

Recently, a paradigm shift toward non-invasive reduction as a bridge to elective surgery has been proposed [8]. In 2024, Kobayashi et al. [7], after analyzing a retrospective cohort through a systematic review involving a total of 112 patients from six studies, compared manual reduction with ultrasound-guided reduction. They found a success rate of 78% for ultrasound-assisted reduction versus 33% for manual reduction. In addition, a lower rate of intestinal resection was observed when the procedure was performed within the first 72 hours. A missed opportunity for our patient.

When non-invasive methods are not feasible, or diagnosis is delayed, the rate of intestinal necrosis increases significantly, reaching 40.7% in cases requiring emergency surgery [6]. In such situations, surgery is imperative. Although laparotomy is the most commonly used technique (76.3%), endoscopic surgery, whether performed using the transabdominal preperitoneal or totally extraperitoneal approach, is gaining ground. In 2025, Baker et al., in a systematic review that included 47 studies, demonstrated that it allows for a shorter hospital stay (three days on average), reduces the risk of recurrence and infection, and lowers mortality compared to open surgery [8]. Furthermore, laparoscopy offers the diagnostic advantage of visualizing occult contralateral hernias, which may be present in a significant percentage of patients, allowing for their simultaneous repair [8,12].

However, laparoscopy requires technical expertise, especially in cases of distended bowel, and may be contraindicated in patients with severe cardiovascular compromise [8]. Due to hemodynamic instability in our patient, it was decided to approach her by laparotomy. The optimal strategy for repairing the obturator defect remains a subject of significant debate, particularly in emergency settings where intestinal viability is compromised.

Primary closure of the obturator canal with non-absorbable sutures has traditionally been the most common approach in emergency surgery. According to Nasir et al. (2012), this technique is safe, quick, and effective, especially when the patient’s stability is a concern [2]. In cases of gross contamination, such as the fecal peritonitis observed in our patient, primary suture repair is often the preferred method to avoid the risks associated with placing a foreign body in an infected field [2,5].

About the use of mesh repair, in 2023, Burla et al. conducted a systematic review with a meta-analysis that included 351 patients, of whom 120 (34.2%) underwent mesh repair, and 231 (65.8%) underwent tension-free repair. A higher relative risk of recurrence was observed in those with mesh-free repair (relative risk (RR) = 0.31; 95% confidence interval (CI) = 0.11-0.94; p = 0.04), with no significant differences in terms of complications (RR = 0.59; 95% CI = 0.28-1.25; p = 0.17; I² = 50%) for both groups [5]. However, its use in the presence of strangulated bowel or perforation is controversial. While some studies suggest that polypropylene mesh can be used safely in “clean-contaminated” cases, Li et al. (2021) found that in the emergency group, where the intestinal necrosis rate reached 40.7%, mesh placement was frequently avoided due to the high risk of prosthetic infection [6].

The mortality rate for complicated obturator hernia remains high, ranging from 10% to 28% [7], and can reach 46.1% in non-surgical cases [6]. In emergency settings requiring bowel resection, which occurs in approximately 40.7% of cases, mortality is significantly higher [13]. Advanced age and a high American Society of Anesthesiologists score are independent predictors of poor outcomes [14]. The fatal outcome in this case underscores that prognosis is determined more by the timing of the diagnosis than by the surgical technique employed [6,12]. Analyzing our case as that of a malnourished patient in shock secondary to sepsis caused by intestinal perforation, it falls into this group with high mortality, consistent with our patient’s clinical outcomes.

## Conclusions

Obturator hernia is a rare but life-threatening condition that requires a high index of clinical suspicion, particularly in elderly, thin women presenting with intestinal obstruction. As demonstrated in this case, the absence of the Howship-Romberg sign and a history of prior abdominal surgeries can lead to diagnostic delays and catastrophic outcomes, such as bowel perforation and fecal peritonitis. The cornerstone of modern treatment is the early use of CT, which is inversely associated with mortality. Although non-invasive reduction and laparoscopic approaches are gaining ground as viable options, they are only feasible in cases of early-stage diagnosis. We conclude that general surgeons should maintain a low threshold for ordering CT scans in geriatric patients to ensure timely intervention and improve survival rates for this surgical emergency.
